# Identification of a Novel Signature Predicting Overall Survival in Head and Neck Squamous Cell Carcinoma

**DOI:** 10.3389/fsurg.2021.717084

**Published:** 2021-09-24

**Authors:** Haige Zheng, Huixian Liu, Yumin Lu, Hengguo Li

**Affiliations:** ^1^Medical Imaging Center, The First Affiliated Hospital of Jinan University, Guangzhou, China; ^2^The First Affiliated Hospital of Guangxi University of Chinese Medicine, Nanning, China

**Keywords:** robust rank aggregation method, signature, nomogram, overall survival (OS), head and neck squamous cell carcinoma (HNSCC)

## Abstract

**Background:** Head and neck squamous cell carcinoma (HNSCC) is a highly heterogeneous tumor with a high incidence and poor prognosis. Therefore, effective predictive models are needed to evaluate patient outcomes and optimize treatment.

**Methods:** Robust Rank Aggregation (RRA) method was used to identify highly robust differentially-expressed genes (DEGs) between HNSCC and normal tissue in 9 GEO and TCGA datasets. Univariate Cox regression analysis and Lasso Cox regression analysis were performed to identify DEGs related to the Overall survival (OS) and to construct a prognostic gene signature (HNSCCSig). External validation was performed using GSE65858 dataset. Moreover, comprehensive bioinformatics analyses were used to identify the association between HNSCCSig and tumor immune environment.

**Results:** A total of 257 reliable DEGs were identified by differentially analysis result of TCGA and GSE65858 datasets. The HNSCCSig including 7 mRNAs (SLURP1, SCARA5, CLDN10, MYH11, CXCL13, HLF, and ITGA3) were developed and validated to identify high-risk group who had a worse OS than low-risk group in TCGA and GSE65858 datasets. Cox regression analysis showed that the HNSCCSig could independently predict OS in both the TCGA and the GSE65858 datasets. Further research demonstrated that the infiltration bundance of CD8 + T cells, B cells, neutrophils, and NK cells were significantly lower in the high-risk group. A nomogram was also constructed by combining the HNSCCSig and clinical characters.

**Conclusion:** We established and validated the HNSCCSig consisting of SLURP1, SCARA5, CLDN10, MYH11, CXCL13, HLF, and ITGA3. A nomogram combining HNSCCSig and some clinical parameters was constructed to identify high-risk HNSCC-patients with poor prognosis.

## Introduction

Head and neck squamous cell carcinoma (HNSCC) ranks sixth among cancer-related deaths worldwide, and includes tumors originating from the mouth, oropharynx, nasopharynx, hypopharynx, larynx, and neck (1). Head and neck cancer is one of the main tumors that threatens human health, with over 600,000 new patients and over 350,000 deaths worldwide every year. Despite the substantial improvement in surgery, radiation therapy and chemotherapy in the past three decades, early diagnosed patients may achieve good results with surgery or radiation therapy, but survival rates for patients with advanced cancer is only 34.9%, and the median survival time of individuals has been reported to be 6 to 9 months (2, 3). Thus, there is an urgent need to identify effective HNSCC predictive biomarkers for an accurate and effective evaluation of a patient's disease status to improve prognosis and reduce mortality.

As we know, the immune system is an important factor in tumorigenesis. As an emerging effective anti-tumor therapy approach, immunotherapy represented mainly by the programmed cell death 1 (PD-1)/programmed cell death ligand 1 (PD-L1) pathway has shown great therapeutic potential in many tumor types (4, 5). Thus, it is important to study the tumor-immunity correlation and provide additional treatment options for HNSCC patients.

With the development of genome-sequencing technology, there is growing evidence that prognostic gene markers can predict head and neck cancer overall survival (OS). For example, Shen et al. (2017) have identified a 7-gene signature associated with the survival of patients with head and neck carcinoma (6). Liu et al. (2018) selected 5-lncRNA and constructed a prognostic score model for the prediction of OS (7). She et al. (2020) predicted the prognosis of HNSCC using immune-related genes (8). However, the small sample size of previous studies and the differences in sequencing platforms may make the research results unreliable. The Robust Rank Aggregation (RRA) method we use can perfectly avoid this issue. It directly integrates the differentially expressed gene lists analyzed by different data sets and identifies a more robust differentially-expressed gene set.

In this study, we aimed to: (I) identify highly robust differentially-expressed genes (DEGs) between HNSCC and normal tissue using RRA method; (II) identify DEGs associated with OS of HNSCC by Cox regression analysis; (III) construct and validate a prognostic gene signature (HNSCCSig) to identify patients with poor OS; and (IV) analyse association between HNSCCSig and tumor immune environment (TME).

## Materials and Methods

### Source of Data

The 10 gene microarray datasets of HNSCC were derived from the GEO database. Nine gene microarray data sets (GSE6631, GSE13601, GSE30784, GSE31056, GSE33205, GSE37991, GSE51985, GSE59102, and GSE138206) were used for DEGs analysis. GSE65858 with 270 tumor tissues and corresponding clinical information were used to verify the HNSCCSig. We directly downloaded the standardized matrix data from the GEO platform and match the probe to the genetic symbol using the manufacturer's annotation file. When a single gene symbol corresponded to several probes, the method of median sequence value was adopted.

HNSCC patient's normalized HTSeq-counts and HTSeq-fpkm data along with corresponding clinical data were obtained from TCGA databases, which contained data from 502 cancer samples and 44 normal samples. The ensemble ID was converted to a gene symbol through the annotation file. HTSeq-counts data were used to screen for DEGs. Finally, the HTSeq-fpkm data were applied to establish a prognostic model, after removing 10 samples with survival time less than one month, Ultimately, 491 samples were incorporated into the modeling analysis.

### Comprehensive Analysis of Data Sets and DEGs Screening

Acquisition of DEGs between HNSCC and adjacent normal tissue were performed using the nine datasets from GEO using “limma” packages from R software (version 3.6.2) (9). The edgeR package was used for DEG analysis of the TCGA queue. The RRA method was used to analyze the DEGs identified from the nine GEO datasets using R package “RobustRankAggreg”. All genes with | LogFC | > 1.0, false discovery rate (FDR) <0.05 and *p* < 0.05 were selected as DEGs. More reliable HNSCC-specific DEGs were obtained by the intersection of nine GEO and TCGA dataset results. The “sva” package was run to eliminate or reduce batch effects between different data sets.

### Functional Analysis of Genes

GO and KEGG enrichment analyses were applied to identify potential biological processes (BPs), cellular components (CCs), molecular functions (MFs), as well as KEGG pathway terms. Important signal pathways related to DEGs were identified using the “clusterprofiler” R package.

### Identification of the Survival-Related Prognostic Model

Univariate, Lasso Cox regression analyses were conducted to obtain prognostic genes significantly correlated with OS in 491 HNSCC patients with survival time >30 days (10). Firstly, Univariate analysis was used to identify genes associated with prognosis. Secondly, Lasso Cox regression analysis was performed to further select candidate genes significantly related to hnscc and the 10-fold cross-validation was used to determine the optimal lambda value, thereby limiting the error to a minimum of 1 standard error. Ultimately, a seven-gene HNSCCSig was established based on LASSO coefficients (β) derived from LASSO model multiplied by the mRNA expression levels.

Using the median value as the cut-off, 491 HNSCC patients were separated into high- and low-risk groups. Kaplan–Meier analysis and receiver operating characteristic curve (ROC) curve analyses were performed to evaluate the accuracy and sensitivity of the model using the R package “survival” and “survivalROC”, respectively (11). The concordance index(C-index) was also used to measure discrimination between the predicted value and the real value of the Cox model.

### GSE65858 Cohort for External Validation

The GSE65858 dataset from the GEO database was used for external validation. Risk scores for each included patient were calculated using the same prognostic gene signature. Next, the predictive capability of HNSCCSig was also tested based on the Kaplan–Meier curve, the ROC curve, and the C-index.

### Independence of Prognostic Genes

Only 378 patients with complete clinicopathological information including age, sex, survival time, survival status, grade, American Joint Committee on Cancer (AJCC) staging, T staging, N staging and M staging were included in our subsequent analysis. Univariate and multivariate analyses were performed to test whether the prognostic risk score could be independent of other clinical variables (such as age, gender, AJCC-stage, T-stage, N-stage, M-stage and grade).

### Establish and Verify a Prognostic Nomogram

A nomogram can be combination with multiple indicators to diagnose or predict disease onset or progression. We constructed the nomogram using T-stage, N-stage, M-stage and risk score to predict the 1-, 3-, and 5-year OS rate of head and neck cancer. Nomogram is generally verified by two indicators, namely, discrimination and calibration. Thus, the nomogram's calibration curves were drawn to compare the actual survival rate and the predicted survival rate, with the ordinate indicating the actual survival rate, and the abscissa indicating the predicted survival rate. While, the C-index was used to determine the discrimination of the nomogram using a bootstrap approach, repeated 1000 times. Kaplan–Meier analysis, Decision Curve Analysis (DCA) curve were also applied to assess the predictability of the prognostic nomogram. DCA was used to predict clinical outcome variables and was performed to quantify the clinical utility of the nomogram and to determine its clinical usefulness (12). The nomogram was used to calculate the total score and patients were separated into two groups. The Kaplan-Meier method was used to plot the survival curves for different risk assessment groups.

### Enrichment and Tumor Immunity Analyses

To clarify the potential pathobiological mechanism underlying the gene signature, GSEA analysis was conducted to identify rich GO terms and KEGG pathways between high-risk and low-risk groups, including the KEGG pathway in C2, GO term in C5, and oncogenic signatures of gene sets in C6.

The stromal, immunity, and estimate scores of each head and neck cancer sample were calculated using the ESTIMATE algorithm. Single-sample Gene set enrichment analysis (ssGSEA) approaches can be used to quantify tumor-infiltrating immune cells using the R package “GSVA” (13). The infiltration abundance of 29 immune cells (including B cells, CD4+ T cells, CD8+ T cells, macrophages, neutrophils, dendritic cells, etc.) in each head and neck cancer sample were calculated. Finally, the correlation between stroma, immunity, estimated score and risk score, and the difference in immune cell infiltration between the high-risk and low-risk groups was analyzed.

### Statistical Analysis

R software (version 3.6.2; http://www.Rproject.org) and GraphPad Prism (v. 8.0) were used for statistical analysis and to prepare figures. The unpaired *t*-test was used to estimate the statistical significance of two groups of normally distributed variables. Kaplan-Meier curve analysis was used for survival analysis, the logarithmic rank test was conducted to assess the survival risk in high and low risk groups. Unless otherwise specified, *p* < 0.05 was considered statistically significant.

## Results

### Screening for Differentially Expressed Genes

**Figure 1:**
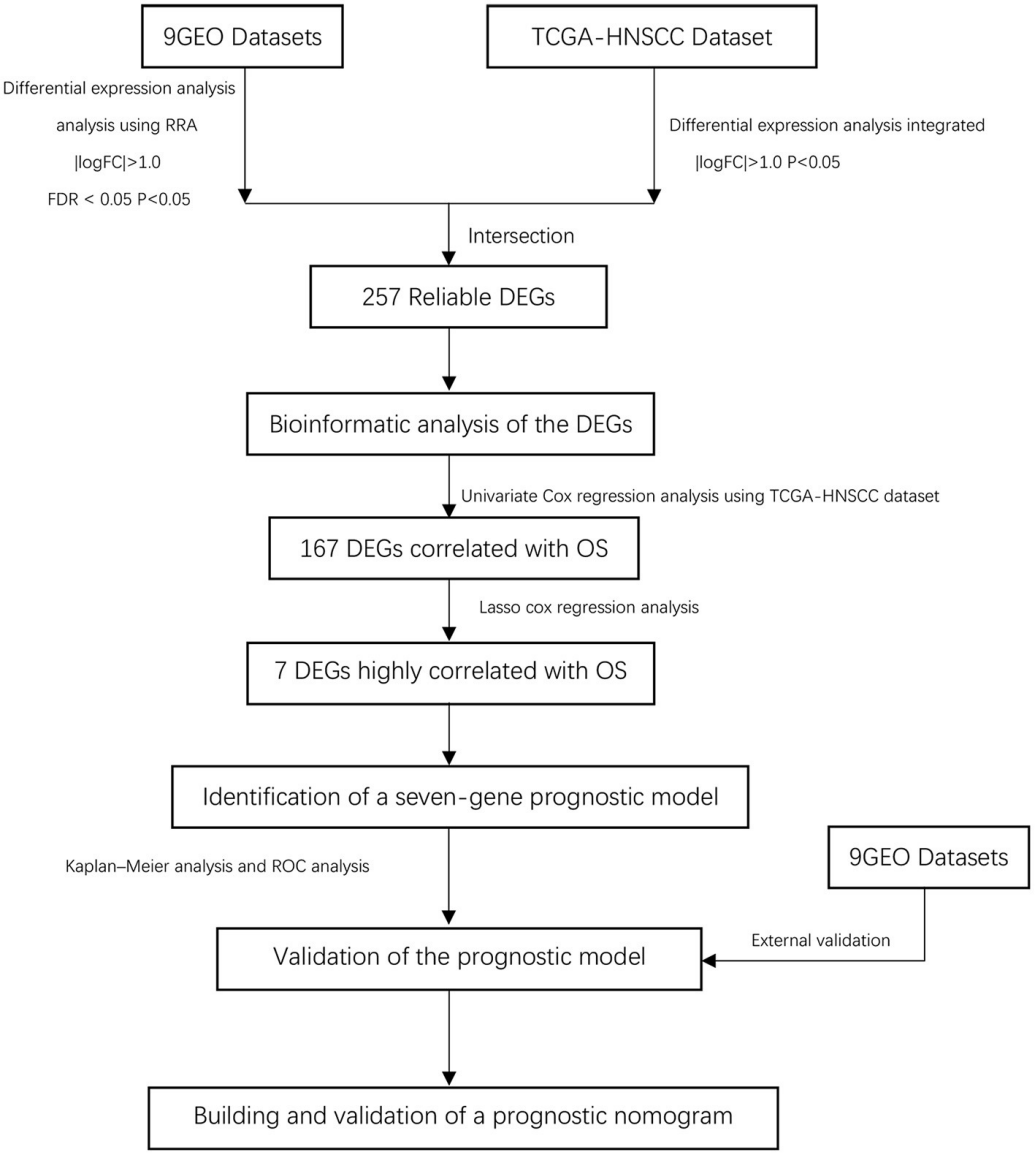
Overall flowchart of this study.

**Figure 2:**
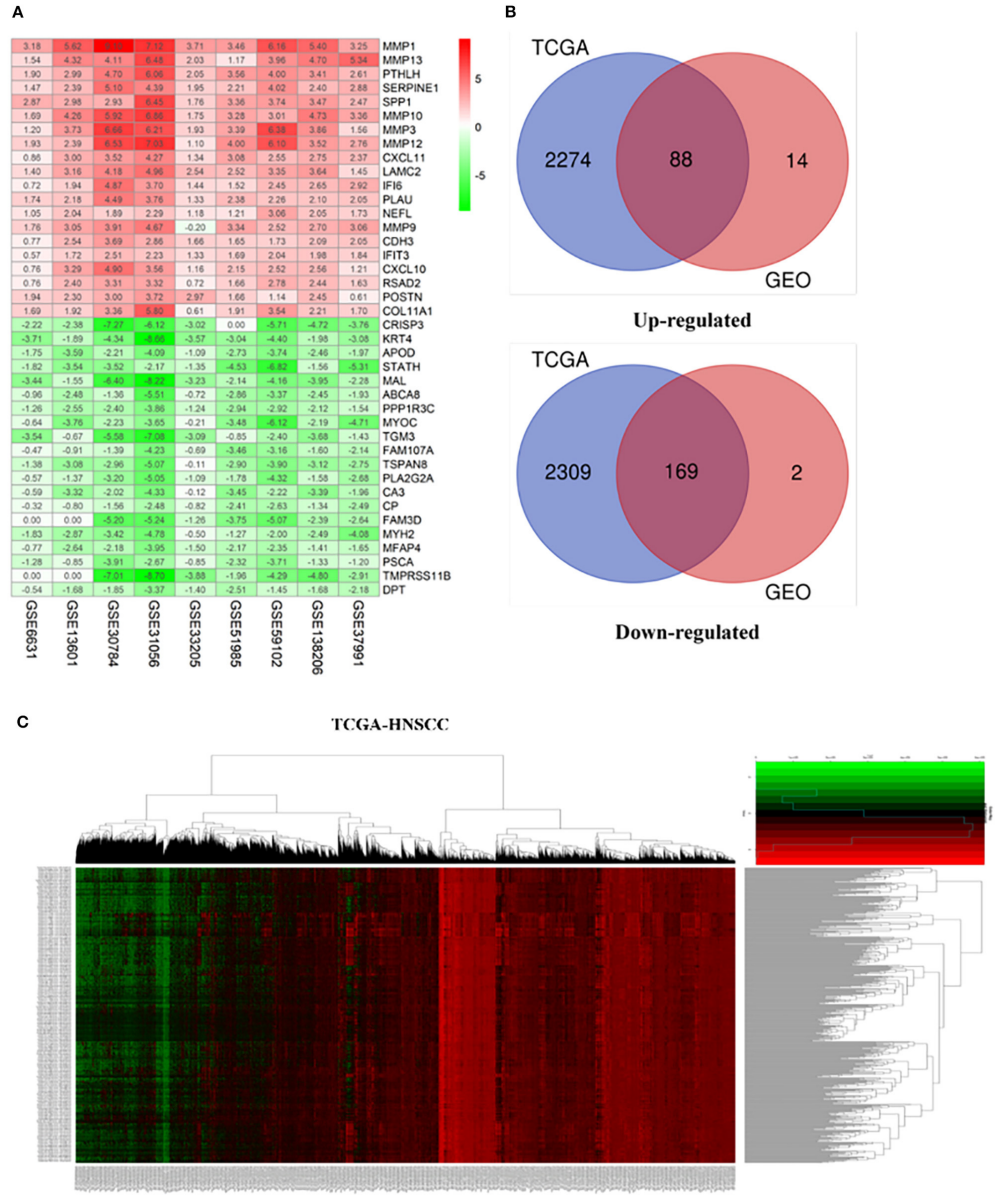
Integrated analysis of differentially expressed genes (DEGs). **(A)** Shows heat maps of the top 20 up-regulated and down-regulated differential genes screened from 9 GEO datasets based on “RobustRankAggre” method. **(B)** Venn diagram of the intersection of TCGA and GEO differential genes. **(C)** The heatmap of differential genes in the TCGA-HNSCC cohort.

Our study follows the flowchart shown in Figure 1. The details of the 10 GEO data sets are shown in Table 1. From the GSE6631, GSE13601, GSE30784, GSE33205, GSE37991, GSE51985, GSE59102 and GSE138206 data sets, 145, 1140, 1582, 2082, 420, 1848, 620, 2758, and 901 differential genes were selected, respectively. After a comprehensive study of nine GSE datasets based on the RRA means, 193 DEGs were identified, including 69 upregulated and 124 downregulated genes. The top 20 upregulated and downregulated DEGs found in the comprehensive analysis were shown in the volcano diagram (Figure 2A). For the TCGA-HNSCC dataset, 3033 DEGs (1359 up-regulated and 1673 down-regulated) were selected, the representative heat map of DEG showed that DEG could effectively distinguish between tumor and normal tissue (Figure 2C). Finally, after the intersection of GEO and TCGA results, 257 reliable DEGs were identified (Figure 2B).

**Table 1:** Details of the GEO datasets included in this study.

**Datasets**	**Platform**	**Sample size (tumor/control)**	**Application**
GSE6631	[HG_U95Av2] Affymetrix Human Genome U95 Version 2 Array	44 (22/22)	Identification of DEGs
GSE13601	[HG_U95Av2] Affymetrix Human Genome U95 Version 2 Array	58 (31/27)	Identification of DEGs
GSE30784	[HG-U133_Plus_2] Affymetrix Human Genome U133 Plus 2.0 Array	212 (167/45)	Identification of DEGs
GSE31056	[HG-U133_Plus_2]Affymetrix Gene Chip HumanGenomeHG-U133Plus2 Array	47 (23/24)	Identification of DEGs
GSE33205	[HuEx-1_0-st]Affymetrix Human Exon 1.0 ST Array [transcript (gene) version]	69 (44/25)	Identification of DEGs
GSE37991	Illumina HumanRef-8 v3.0 expression bead chip	80 (40/40)	Identification of DEGs
GSE51985	Illumina HumanHT-12 V4.0 expression bead chip	20 (10/10)	Identification of DEGs
GSE59102	Agilent-014850 Whole Human Genome Microarray 4x44K G4112F	42 (29/13)	Identification of DEGs
GSE138206	[HG-U133_Plus_2] Affymetrix Human Genome U133 Plus 2.0 Array	18 (12/6)	Identification of DEGs
GSE65858	Illumina HumanHT-12 V4.0 expression bead chip	270	Validation of prognostic gene

### Functional Analysis of Genes

**Figure 3:**
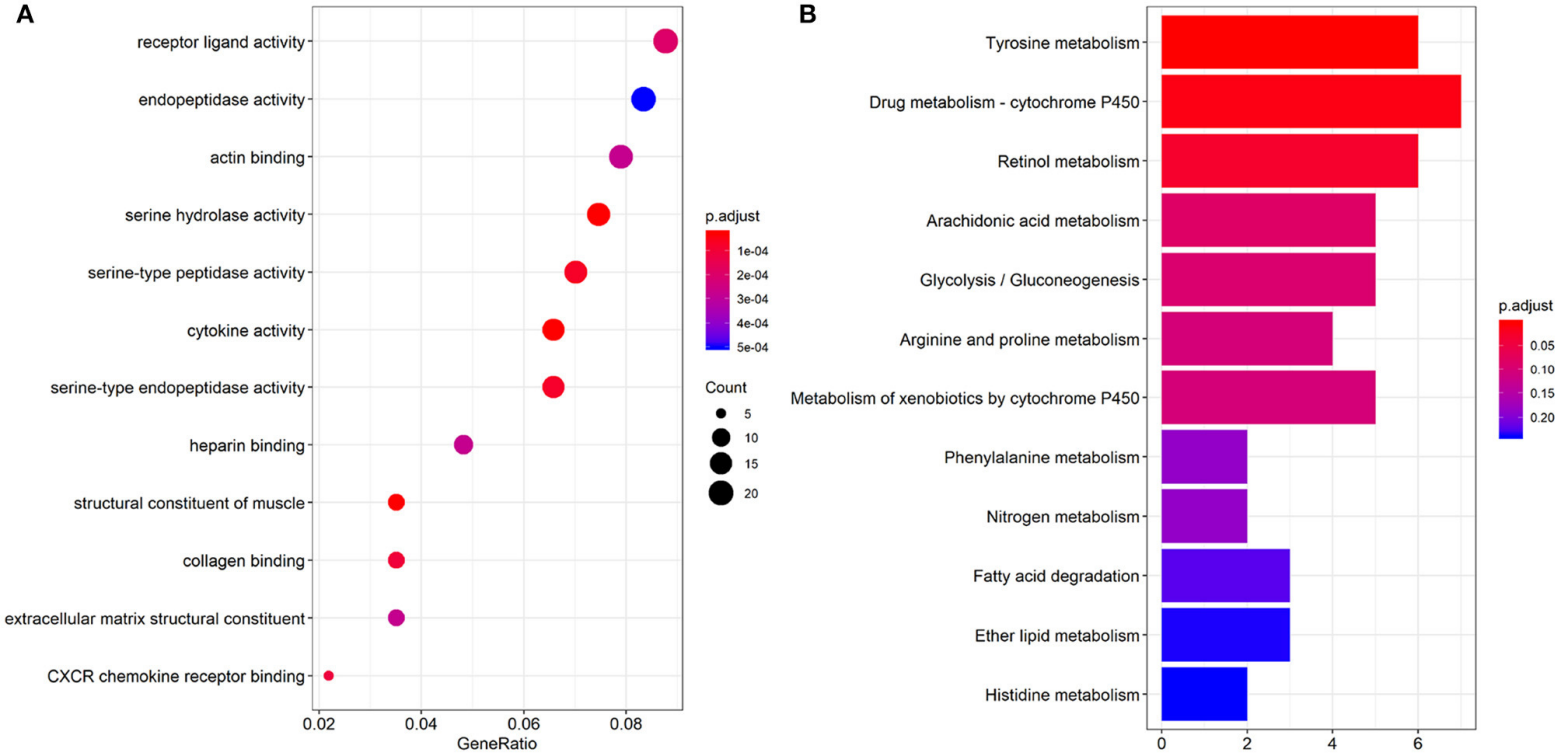
Results of GO function **(A)** and KEGG pathway enrichment analyses **(B)**.

GO enriched sets (*p* < 0.0005) were analyzed relative to genes involved in serine hydrolase activity, cytokine activity, muscle structure component, serine type peptidase activity, serine-type endopeptidase activity, collagen binding, CXCR chemokine receptor binding, receptor-ligand activity, actin-binding, heparin-binding, extracellular matrix structural constituent (Figure 3A). The KEGG pathway analysis of the differential genes mainly involved 3 pathways (*p* < 0.05): tyrosine, retinol metabolism, and drug metabolism cytochromes (Figure 3B).

### Establishment of a Prognostic Model

**Figure 4:**
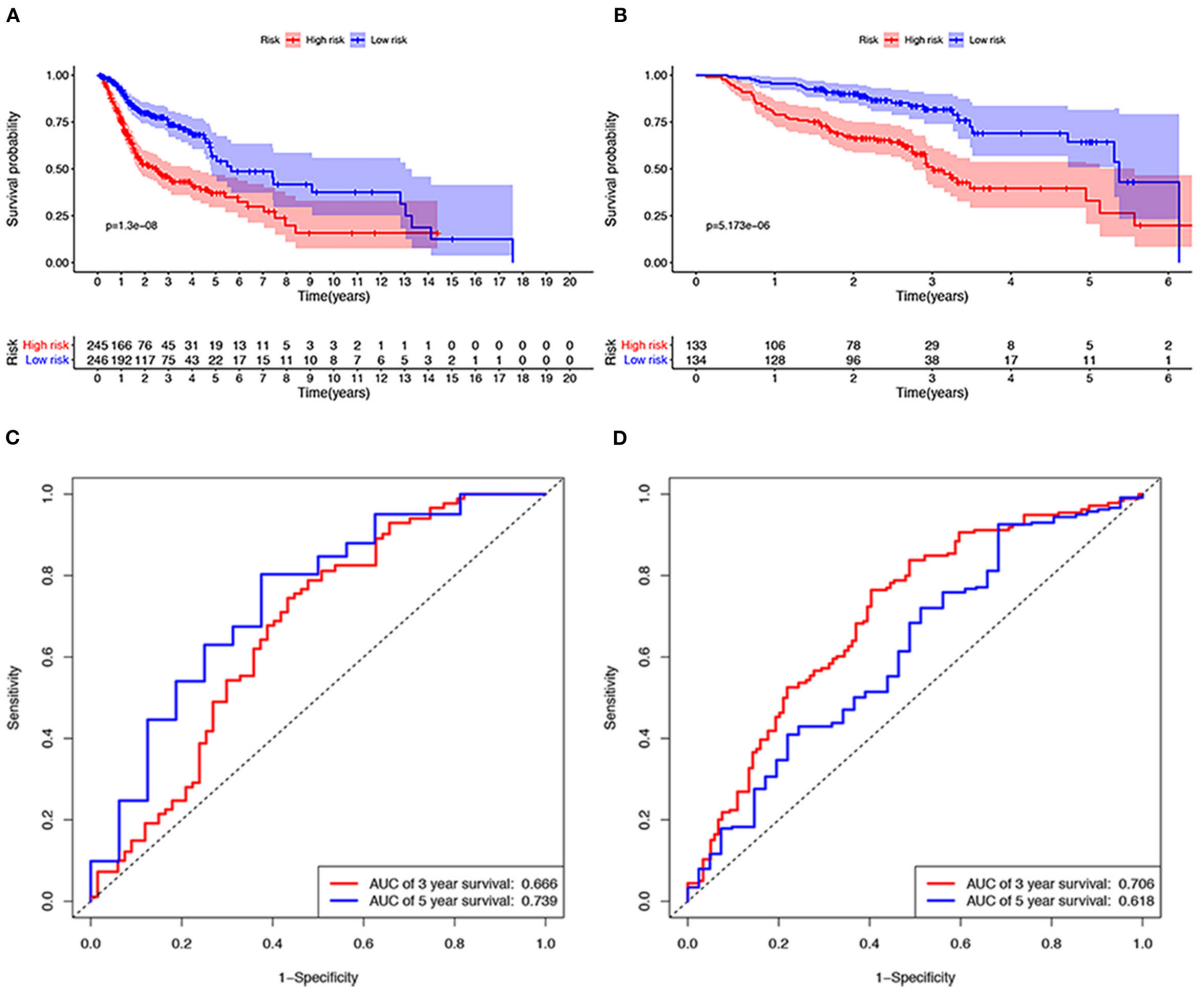
Verification of the seven gene signatures in training and validation cohort. Kaplan–Meier analysis shows that patients with high risk scores have poorer OS, whether in the TCGA (**A**) or the GSE65858 data set **(B)**. The ROC curve shows the accuracy of predicting the 3-year and 5-year OS of patients in the TCGA **(C)** and GSE65858 data sets **(D)**.

A total of 491 TCGA patients with a survival greater than one month were incorporated into this model analysis. The basic clinical parameters of the included patients are shown in Table 2. Univariate analysis identified 166 genes that were significantly associated with OS. Through Lasso Cox regression analysis, then, seven genes were selected to build a predictive HNSCCSig. The formula for the risk score is as follows: risk model = (−0.00173 ^*^ SLURP1) + (−0.21582 ^*^ SCARA5) + (−0.0458 ^*^ CLDN10) + (0.030114 ^*^ MYH11) + (−0.00725 ^*^ CXCL13) + (−0.0839 ^*^ HLF) + (0.005721^*^ ITGA3). Next, Kaplan–Meier analysis disclosed a significantly better prognosis in the low-risk group (*P* < 0.0001) (Figure 4A). In addition, the ROC and C-indexes also evaluated the good predictive power of the HNSCCSig. The time-dependent AUCs of risk scores were 0.666 at 3-year and 0.739 at 5-year OS (Figure 4C). The C-index of the risk score was 0.623 (95% CI, 0.538–0.708).

**Table 2:** Patients' information of this study from the TCGA and GEO databases.

**Characteristic**		**TCGA training datasets (*n* = 491)**	**GSE65858 (*n* = 270)**
**Age (years)**	< =50	87	47
	>50	404	223
**Survival Status**	Living	302	176
	Dead	189	94
**Gender**	Female	130	47
	male	361	223
**Grade**	G1	60	\
	G2	293	\
	G3	117	\
	G4	2	\
	GX	19	\
**PathologicT**	T1	44	35
	T2	129	80
	T3	96	58
	T4	166	97
	Unknow	56	\
**PathologicN**	N0	167	94
	N1	65	32
	N2	159	132
	N3	7	12
	NX/unknow	93	\
**PathologicM**	M0	366	263
	M1	12	7
	MX	113	\
**Tumor stage**	Stage I	25	18
	Stage II	69	37
	Stage III	78	37
	Stage IV	251	178
	Unknow	68	\

### External Validation Set and Performance

The GSE65858 dataset with 267 HNSCC patients was selected to validate the HNSCCSig. We calculated risk scores with the same risk formula and divided the patients in the GSE65858 dataset into high-risk and low-risk groups. The model established in this study was also meaningful in the validation set, the results show that significant differences in OS between high- and low-risk groups (*p* < 0.0001) (Figure 4B). The predicted AUC for the 3- and 5-year OS were 0.706 and 0.618, respectively, indicating good diagnostic performance (Figure 4D). The C-index of the gene signature was 0.641(95% CI, 0.528 – 0.754).

### Subgroup Analysis on the Seven-Gene Signature

**Figure 5:**
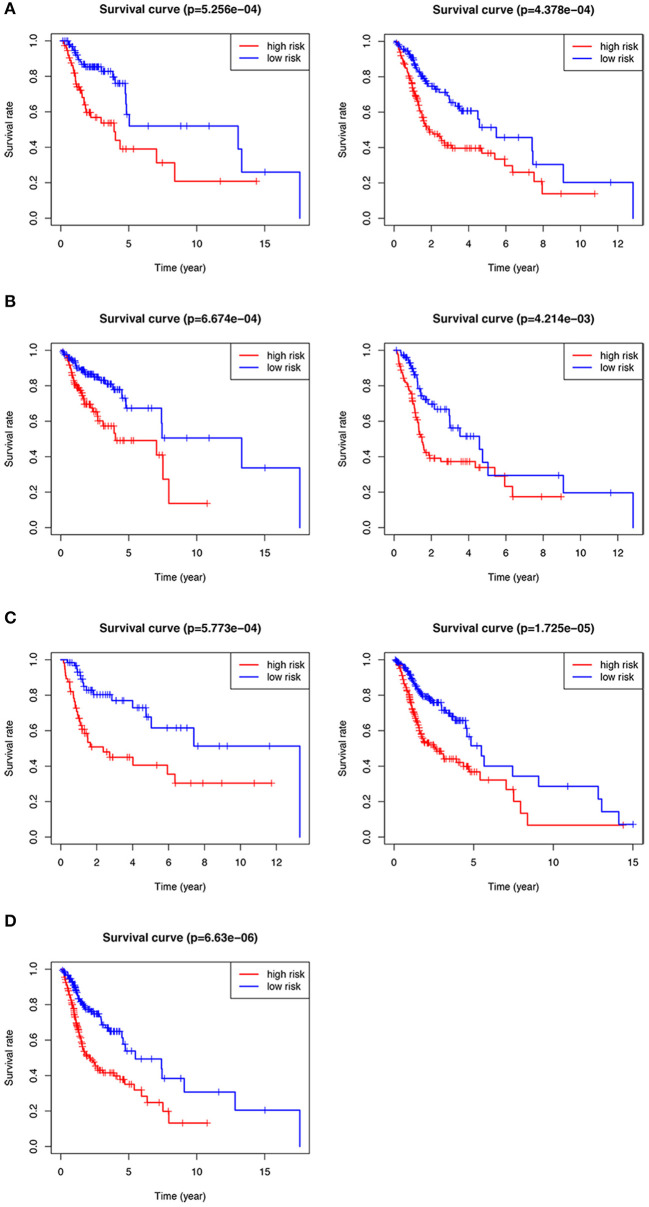
Kaplan–Meier analysis of different subgroups in the TCGA test set. **(A)** T1-2 & T3-4 patients. **(B)** N0-1 & N2-3 patients. **(C)** G1-2 & G3-4 patients. **(D)** Stage III-IV patients.

We performed subgroup analysis on the TCGA data set to further verify the validity of HNSCCSig and the results revealed that the model also had certain diagnostic performance in different subgroups (Figures 5A–D). They were stage T1-2 patients (*p* < 0.001), T3-4 patients (*p* < 0.001), N0-1 patients (*p* < 0.001), N2-3 patients (*p* = 0.004), G1-2 patients (*p* < 0.001), G3-4 patients (*p* < 0.001), and stage III-IV (*p* < 0.001).

### Independent Predictability of Prognostic Models

**Figure 6:**
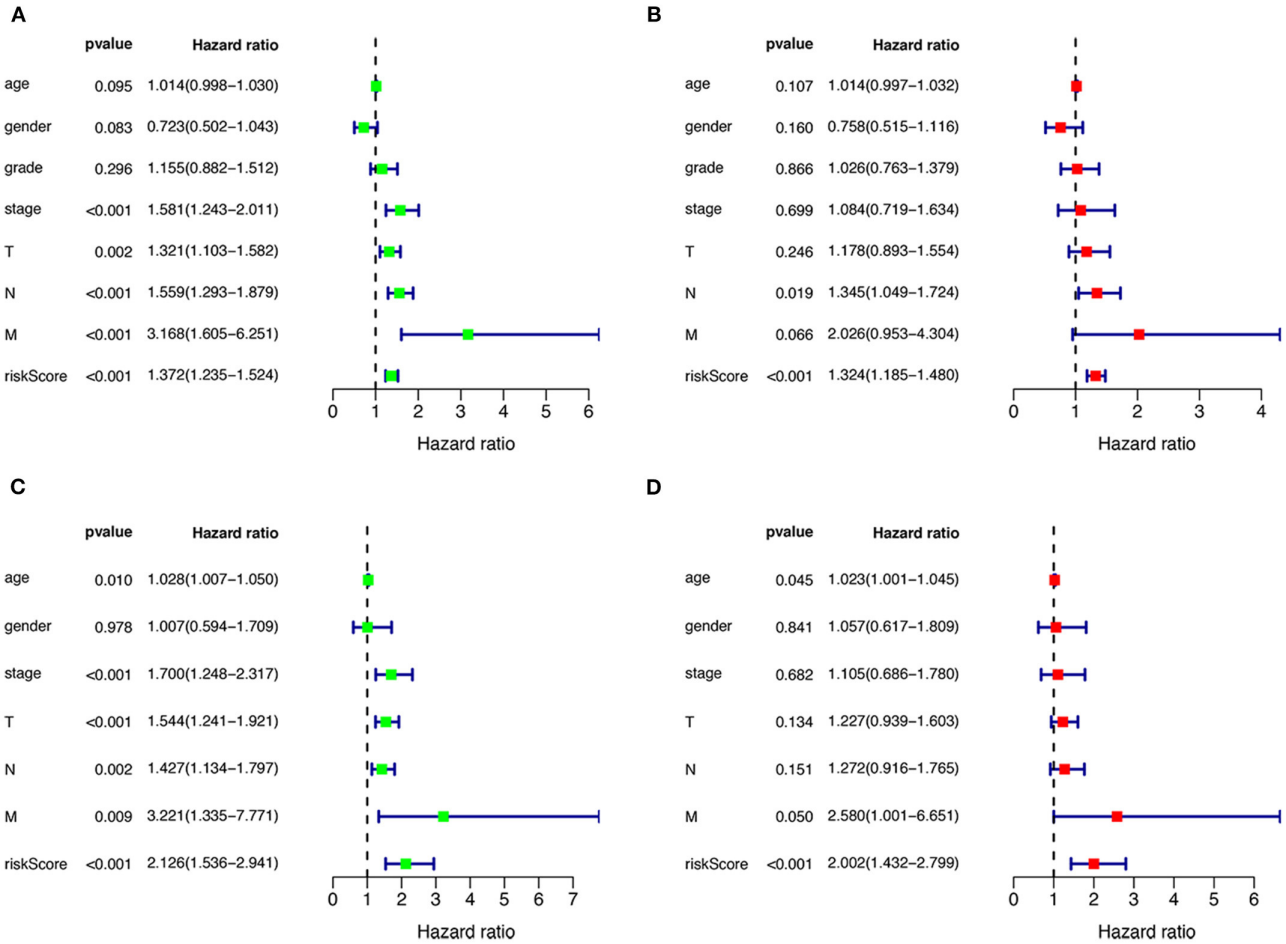
Cox regression analysis to detect clinical independence of risk scores. Univariate **(A)** and Multivariate analysis **(B)** indicated that the risk score is an independent predictor variable in the TCGA dataset**. (C, D)** The Cox regression analysis also shows that the risk score can be independent of other clinical variables in the validation set GSE65858.

Univariate and multivariate Cox regression were performed to assess the independent predictive capacity of the seven genes HNSCCSig based on 378 HNSCC patients with available clinicopathological parameters. The *p*-value for Stage, T-stage, N-stage, M-stage and the riskscore was <0.05 in univariate Cox regression analysis (Figure 6A). Next, multivariate Cox regression analysis demonstrated that N-stage and the riskscore can independently predict OS for head and neck cancer (Figure 6B). The same approach was used for the validation set, and results also indicated that the risk score was an independent prognostic factor (Figures 6C,D).

### Establishment and Verification of the Nomogram

**Figure 7:**
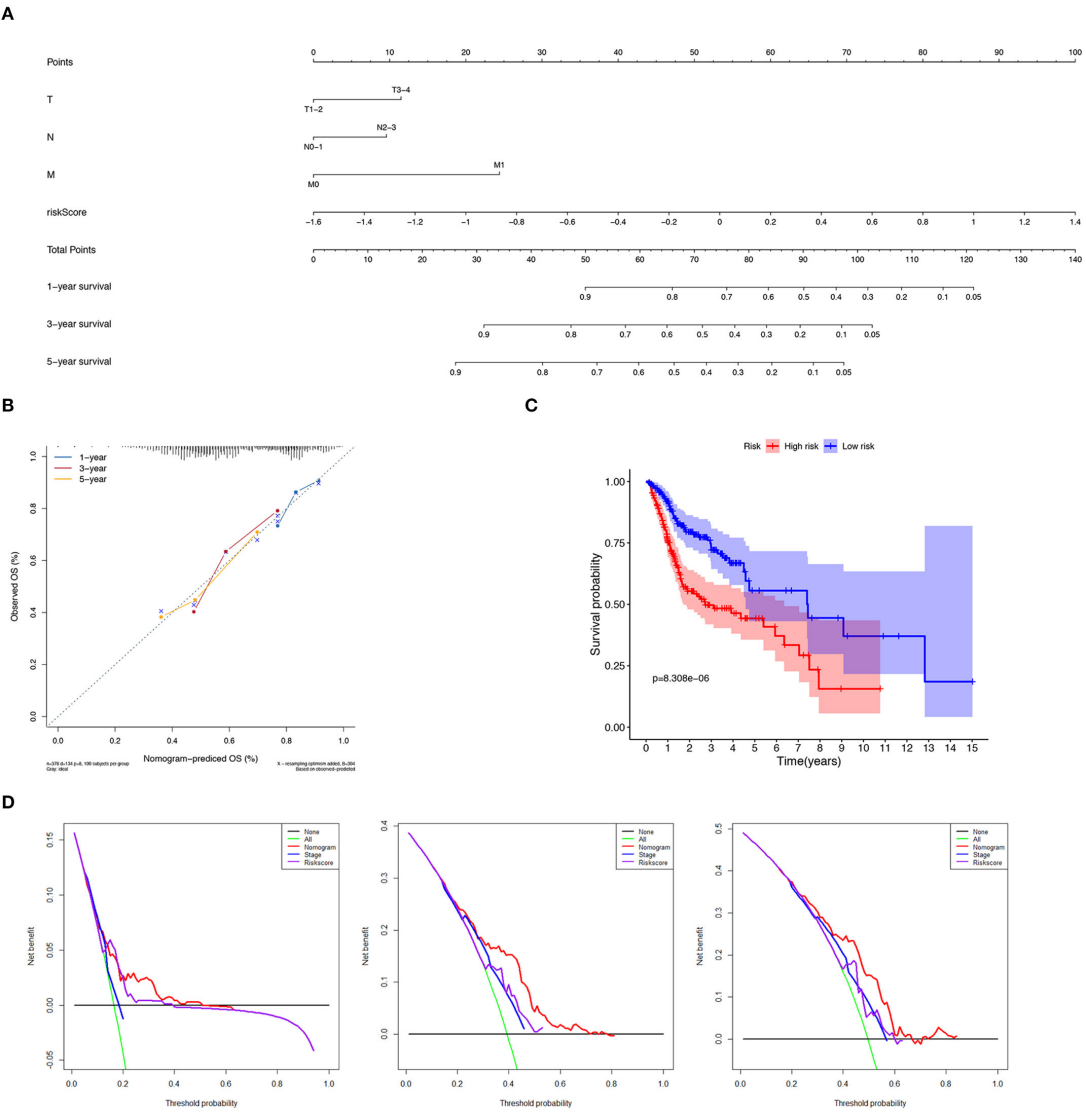
Verification of nomogram prediction performance. **(A)** A nomogram based on 7 genes and related clinical parameters. **(B)** The calibration curve reflects the accuracy of the nomogram to estimate the risk. **(C)** The Kaplan–Meier analysis of the nomogram. **(D)** The DCA curves evaluate the clinical benefit and the application range of the nomograms.

We constructed a nomogram to predict 1-, 3-, and 5-year OS of HNSCC patients based on the TCGA-HNSCC cohort using prognostic factors including T-stage, N-stage, M-stage and risk score (Figure 7A). The C-index of the nomogram was 0.728 (95% CI, 0.568–0.734). The calibration curve intuitively demonstrated that the predicted value of the nomogram was close to the true value and had reliable prediction performance (Figure 7B). The group with a lower risk score had a significantly better prognosis (Figure 7C). The DCA results further suggested that the nomogram was more clinically useful than the gene-based risk model or staging system alone (Figure 7D).

### Gene Set Enrichment Analysis

**Figure 8:**
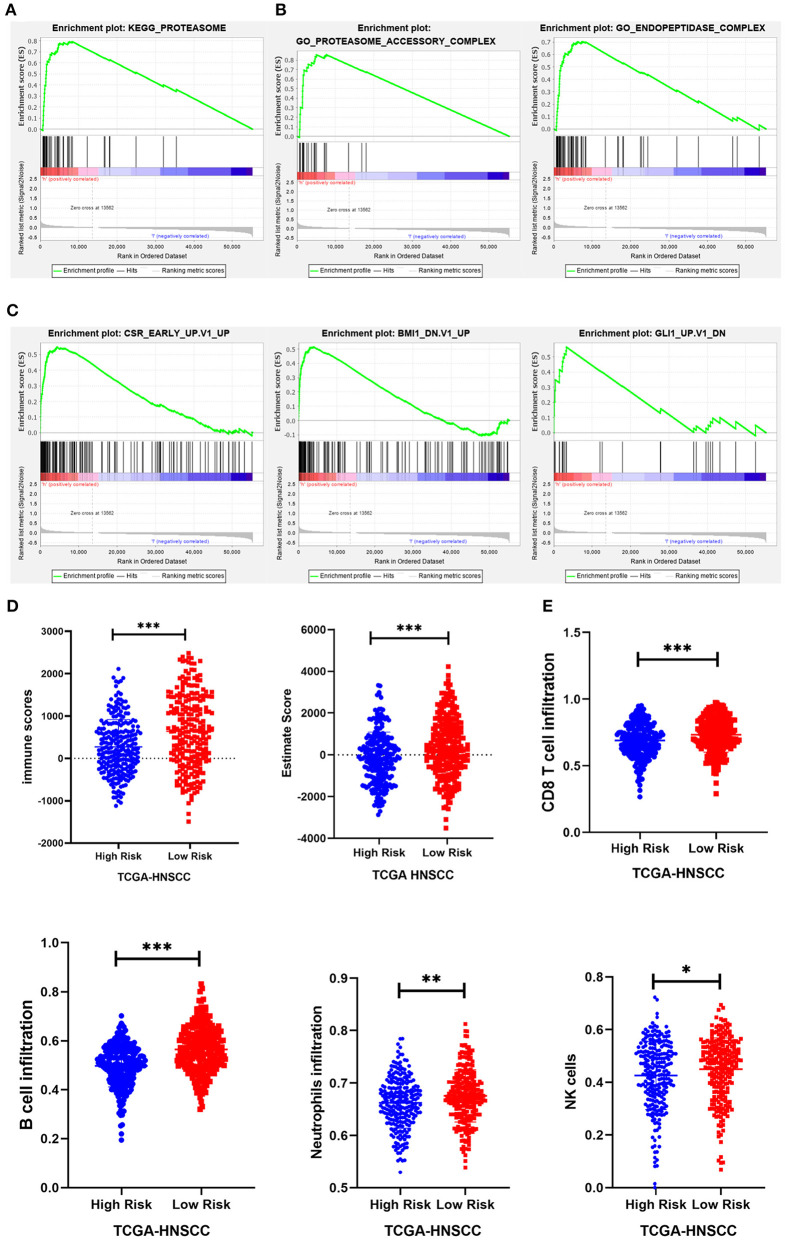
GSEA and immunity correlation analysis of seven prognostic genes. **(A–C)** The results showed that 1 KEGG pathway, 2 GO terms, and 3 oncological signatures were enriched in the high-risk group. **(D)** The immune score and estimated score of the high-risk group were significantly lower in the TCGA cohort. **(E)** The infiltration level of immune cells (including CD8+ T cells, B cells, neutrophils, and NK cells) in the low-risk group was significantly higher than that in the high-risk group in the TCGA cohort. *P < 0.05, **P < 0.01, and ***P < 0.001 respectively.

To investigate the possible underlying pathobiological mechanisms of the prognostic genes, Gene Set Enrichment Analysis (GSEA) was used to analyze important enrichment pathways in different risk groups of TCGA. A total of 491 TCGA-HNSCC patients were enrolled in the GSEA comparison high-risk and low-risk cohort. The results indicated that 1 KEGG pathway, 2 GO terms, and 3 oncological signatures were enriched in the high-risk group. The identified enriched KEGG pathway and GO terminology mainly involved the proteasome pathway, proteasome accessory and endopeptidase signal transduction pathway, and three oncological signatures included early serum response (CSR), glioma-associated oncogene (GLI1) and B cell-specific Moloney murine leukemia virus integration site (BMI1) (Figures 8A–C).

### Tumor Immune Mechanism of Prognostic Genes

Expression data obtained from the TCGA HNSCC dataset were used to calculate the matrix, immunity, and estimated scores using an estimation algorithm. The results revealed that the immunity scores and estimated scores of the high-risk group were significantly lower, indicating that there was less infiltration of immune cells in the cancerous tissue (*p* < 0.05; Figure 8D). The difference in the matrix score between the different risk groups was not statistically significant (*p* > 0.05). To further study the relationship between the seven prognostic genes and immune infiltration in head and neck carcinoma, we used the ssGSEA method to assess the correlation between these genes and the level of immune cell infiltration, including CD4+ T, CD8+ T, B cell, dendritic cells, neutrophils, macrophages and other immune cells. The results suggested that there was a relatively higher level of CD8+ T cells, B cells, neutrophils, and NK cell infiltration in the low-risk group (Figure 8E).

## Discussion

HNSCC is a highly heterogeneous tumor with a very poor prognosis and its incidence is increasing every year. The five-year survival rate is only 40–50% (2). With the expanding research in the field of gene sequencing technology, gene expression profiling has attracted increasing attention, and has been used to identify prognostic markers associated with the heterogeneity of tumors. Therefore, screening for prognostic molecular markers that fully reflect the tumor's biological characteristics may provide clinicians with novel tools to treat HNSCC patients.

In this study, we screened DEGs in head and neck cancer tissues and adjacent non-tumor tissues, and performed Univariate, Lasso Cox analysis to establish HNSCCSig. We identified seven DEGs: SLURP1, SCARA5, CLDN10, MYH11, CXCL13, HLF, and ITGA3, and among them MYH11 and ITGA3 have defined risk factors and the remainder as protective factors. Five of the seven gene signatures have previously been linked to head and neck cancer.

SCARA5, a member of the scavenger receptor Type A family, can bind lipopolysaccharide, bacteria, and nucleotides to charge residues in the cysteine domain. In addition, the SCARA5 receptor can recognize and engulf pathogens, and then transmit intracellular signals to generate an immune response. When the number of receptors is reduced, the cell is unable to mount an immune-induced defense. The reduction of SCARA5 expression can promote invasiveness and proliferation of oral tumor cells, and the expression of SCARA5 was found to be down-regulated in oral tumors (14). SCARA5 expression is also usually downregulated in hepatocellular carcinoma due to high promoter methylation and allele imbalance (15).

MYH11, a smooth muscle myosin, may be involved in cell migration and adhesion, intracellular transport, and signal transduction, and as a contractile protein, it converts the chemical energy hydrolyzed by adenosine triphosphate (ATP) into mechanical energy (16). MYH11 is closely related to the survival of HNSCC, acute myeloid leukemia, colorectal cancer, bladder cancer, and other tumors (17–19).

CXCL13 is an independent and cloned B lymphocyte chemokine named Angie, which is an antimicrobial peptide. CXC chemokines are highly expressed in spleen follicles, lymph nodes, and Peyer's patches. It promotes B-cell migration by stimulating chemotaxis into cells expressing Burkitt lymphoma receptor 1(Blr-1) and calcium influx (20). Previous studies have shown that CXCL13 is associated with the prognosis of various cancers. For example, oral squamous cell carcinoma, breast cancer, and prostate carcinoma (21–24).

Hlf-encoded proteins are a member of the proline and acid-rich (PAR) protein family, which activate transcription by forming homotypic or heterotypic dimers with other PAR family members and binding specific promoter elements. It has been reported that HLF is closely related to the prognosis in Oral Cancer, liver cancer, gastric cancer, and lung cancer among others (25–28).

The ITGA3-encoded protein belongs to the integrin family. Integrin is an isomeric membrane protein composed of chains and chains, which acts as a cell surface adhesion molecule. the down-regulation of ITGA3 reduces the phosphorylation of AKT, extracellular signal-regulated kinases 1 and 2 (ERK1/2), and focal adhesion kinase (FAK) in SAS cells and significantly inhibited migration of cancer cells and invasion of HNSCC cells. High expression of ITGA3 predicted poor survival in patients with HNSCC (29). Moreover, several studies have confirmed that ITGA3 is a marker of glioblastoma, pancreatic cancer, and thyroid cancer (30–32).

The role of SLURP1 and CLDN10 in head and neck cancer has not been described. The protein encoded by SLURP1, a member of the Ly6/uPAR family, has anti-tumor activity (33). SLURP1 is related to the occurrence and development of pancreatic cancer. First, it can not only reduce invasion of cancer cells by controlling AKT, ERK, and NF-kB signaling, but also attenuates nicotine-mediated migration and invasion possibly through competing binding sites (34). Furthermore, mutations in SLURP1 can increase the incidence of melanoma and mucosal skin cancer (35).

Studies have shown that CLDN10 is highly expressed in thyroid papillary carcinoma, and can affect cell proliferation, migration, and invasion *in vitro*; further, it plays the role of a tumor promoter, and the up-regulation of CLDN10 is related to lymph node metastasis (36). In contrast, low expression of CLDN10 indicated a poor prognosis in lung cancer patients. The expression of CLDN10 was negatively correlated with the expression of c-fos. c-fos is considered a recognized oncogene, CLDN10 may control the invasion and metastasis of lung cancer cells by inhibiting the c-fos pathway (37). In our study, the down-regulation of CLDN10 was associated with poor survival rates for HNSCC, which was consistent with the latter view. The mechanism of SLURP1 and CLDN10 in head and neck tumors deserves further study.

Many studies have shown that the immune system acts to control tumor growth and progression, and the prognosis of the tumor is related to lymphocyte infiltration. As an emerging anti-tumor force, immunotherapy has shown great therapeutic potential in many cancers, HNSCC is no exception. In addition, HNSCC patients with highly infiltrated CD8 T cells have a better prognosis, especially in patients who are HPV positive (38). Our results also suggest that the infiltration levels of CD8+ T cells, B cells, neutrophils, and NK cells are lower in the high-risk group.

Our research has many advantages. Firstly, the number of samples was more than in previously published studies, we integrated 9 GEO data sets and TCGA data sets based on the RRA method, which obtain more reliable differential genes. Secondly, we use the Lasso analysis method to establish HNSCCSig to prevent the model from overfitting. HNSCCSig has good predictive accuracy and is verified by an external cohort. Furthermore, C-index, Calibration curves, Kaplan–Meier analysis, DCA curve were applied to assess the predictability of the prognostic nomogram. Finally, we also analyzed the relevance between prognostic gene expression and the immune microenvironment. Nonetheless, the present study also includes certain limitations. First, this study only included clinical parameters such as age, gender, grade, AJCC-staging, T staging, N staging and M staging, and because the TCGA database lacks other host-related factors and complete histopathological variables. Second, the results obtained by bioinformatics analysis alone are not sufficient and need to be confirmed by experimental verification. Therefore, further identification of prognostic biomarkers requires experimental studies with larger samples and experimental validation.

## Conclusion

In conclusion, we established and validated the HNSCCSig consisting of SLURP1, SCARA5, CLDN10, MYH11, CXCL13, HLF, and ITGA3. Our results suggested that the seven genetic markers were closely related to the prognosis and progression of HNSCC, both in the training set and the validation set. A nomogram combining HNSCCSig and some clinical parameters was construct to identify high-risk HNSCC patients with poor prognosis. Notably, our research shows that the high-risk group has a low degree of immune cell infiltration, which may provide new insights into the research of hnscc's immune microenvironment.

## Data Availability Statement

The datasets presented in this study can be found in online repositories. The names of the repository/repositories and accession number(s) can be found in the article/supplementary material.

## Ethics Statement

Ethical review and approval was not required for the study on human participants in accordance with the local legislation and institutional requirements. Written informed consent for participation was not required for this study in accordance with the national legislation and the institutional requirements.

## Author Contributions

HZ: study design and the drafting of the manuscript. HZ and HLiu: data acquisition, analysis, and manuscript modification. HZ, YL, and HLi: the research oversight and manuscript reviews. All authors contributed to the article and approved the submitted version.

## Conflict of Interest

The authors declare that the research was conducted in the absence of any commercial or financial relationships that could be construed as a potential conflict of interest.

## Publisher's Note

All claims expressed in this article are solely those of the authors and do not necessarily represent those of their affiliated organizations, or those of the publisher, the editors and the reviewers. Any product that may be evaluated in this article, or claim that may be made by its manufacturer, is not guaranteed or endorsed by the publisher.
